# Analysis of microbial diversity and functional differences in different types of high‐temperature Daqu

**DOI:** 10.1002/fsn3.2068

**Published:** 2020-12-17

**Authors:** Yurong Wang, Wenchao Cai, Wenping Wang, Na Shu, Zhendong Zhang, Qiangchuan Hou, Chunhui Shan, Zhuang Guo

**Affiliations:** ^1^ Hubei Provincial Engineering and Technology Research Center for Food Ingredients Hubei University of Arts and Science Xiangyang China; ^2^ School of Food Science Shihezi University Shihezi China; ^3^ Hubei Yaozhihe Chuwengquan Liquor Industry Co., Ltd. Xiangyang China

**Keywords:** bacterial diversity, Chinese Maotai‐flavor liquor, functional prediction, high‐temperature Daqu, Illumina MiSeq high‐throughput sequencing

## Abstract

Bacterial communities that enrich in high‐temperature Daqu are important for the Chinese maotai‐flavor liquor brewing process. However, the bacterial communities in three different types of high‐temperature Daqu (white Daqu, black Daqu, and yellow Daqu) are still undercharacterized. In this study, the bacterial diversity of three different types of high‐temperature Daqu was investigated using Illumina MiSeq high‐throughput sequencing. The bacterial community of high‐temperature Daqu is mainly composed of thermophilic bacteria, and seven bacterial phyla along with 262 bacterial genera were identified in all 30 high‐temperature Daqu samples. Firmicutes, Actinobacteria, Proteobacteria, and Acidobacteria were the dominant bacterial phyla in high‐temperature Daqu samples, while *Thermoactinomyces*, *Staphylococcus*, *Lentibacillus*, *Bacillus*, *Kroppenstedtia*, *Saccharopolyspora*, *Streptomyces,* and *Brevibacterium* were the dominant bacterial genera. The bacterial community structure of three different types of high‐temperature Daqu was significantly different (*p* < .05). In addition, the results of microbiome phenotype prediction by BugBase and bacterial functional potential prediction using PICRUSt show that bacteria from different types of high‐temperature Daqu have similar functions as well as phenotypes, and bacteria in high‐temperature Daqu have vigorous metabolism in the transport and decomposition of amino acids and carbohydrates. These results offer a reference for the comprehensive understanding of bacterial diversity of high‐temperature Daqu.

## Graphical abstract

Thermoactinomyces, Staphylococcus, Lentibacillus, Bacillus, Kroppenstedtia, Saccharopolyspora, Streptomyces, and Brevibacterium were the dominant bacterial genera. Bacterial community structure of white Daqu samples and yellow Daqu samples may be more similar, while that of black Daqu samples differs more from the two. Bacteria from different types of high‐temperature Daqu have similar functions as well as phenotypes, and bacterial metabolism was vigorous in the high‐temperature Daqu.

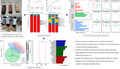

## 1 INTRODUCTION

Traditional Chinese liquor has a long history and wide varieties, its style and flavor are also diversified. Maotai‐flavor liquor is one of the most complex and typical liquor‐flavor types in traditional Chinese liquor, which is popular among consumers. The production of maotai‐flavor liquor belongs to traditional brewing and is a spontaneous solid‐state bilateral fermentation process with multiple strains (Du et al., 2019; Khan et al., 2016; Liu et al., 2019). The fermentation yield and quality are closely related to the microorganisms in the brewing process (Yang et al., 2018). The microorganisms in maotai‐flavor liquor brewing mainly come from high‐temperature Daqu (HTD), the saccharifying and fermenting agent used in maotai‐flavor liquor brewing process (Li et al., 2017; Yang et al., 2018). HTD is made from wheat, barley, and pea as main raw materials through shaping, fermenting, and ripening, and contains a large number of microorganisms and enzymes (Du et al., 2019; Khan et al., 2016; Li et al., 2017). It is a direct factor affecting the yield and liquor quality of base liquor (Liu et al., 2019; Wang et al., 2008). HTD of maotai‐flavor liquor has a complex microbial community structure, mainly including yeasts, bacteria, molds, and a small number of actinomycetes, which come from raw materials, mother Daqu and production environment (Du et al., 2019; Su et al., 2015). The fermentation temperature in the process of HTD making is as high as 60–70°C, so that the succession of microbial community structure and the effective enrichment of enzyme in HTD can be achieved, thus achieving the function of raw material degradation and metabolic fermentation (Wang et al., 2019; Xie et al., 2020). In special HTD‐making environment, the HTD of maotai‐flavor liquor forms a unique microbial community structure through fermentation. With the increase in HTD‐making temperature, most of the temperature‐intolerant yeasts and molds were eliminated, and the microbial community structure of HTD mainly began to propagate bacteria, forming a special microbial community structure dominated by thermophilic bacteria (Gan et al., 2019; Li et al., 2014; Xie et al., 2020; Yang et al., 2018). Moreover, bacteria are also important functional microbial community in HTD, which can metabolize a variety of enzymes and produce abundant liquor aroma substances along with their precursors, endowing the liquor body with a highly complex, sweet, and refreshing flavor of maotai style (Wang, Wu, et al., 2017; Wang, Ban, et al., 2017; Xiao et al., 2017; Xie et al., 2020; Yan et al., 2019; Zhao et al., 2020). As a result, a systematic study on the diversity and function of bacterial communities in HTD is the premise and basis for fully understanding its brewing microbial resources, analyzing the fermentation mechanism of sauce‐flavor liquor and improving the product quality.

**FIGURE 1:**
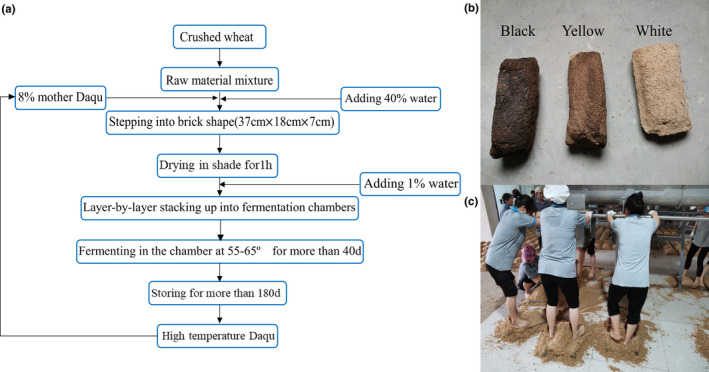
The workflow traditional method for producing HTD (a); three different types of HTD, from left to right, are as follows: black Daqu, yellow Daqu, and white Daqu (b); the barefoot stepping process by female workers (c)

In addition, the abundant microorganisms and complex enzymes in HTD result in considerable Maillard reactions during the HTD‐making process, after which, Maillard reaction causes browning and maotai‐flavoring effects of HTD (Gan et al., 2019; Xie et al., 2020). In the process of HTD making, due to the difference in temperature, moisture, and other parameters among the different locations of HTD stacked in the fermentation chamber, the degree of browning caused by Maillard reaction of high‐temperature Daqu is also different (Li, Bai, et al., 2014). According to the color of HTD, it is generally divided into three types, namely white Daqu, black Daqu, and yellow Daqu (Figure 1b). White Daqu is also named underfermented Daqu, which is generally distributed at the top layer of the Daqu stack and accounts for 10%‐15% of the total HTD; black Daqu is also called overfermented Daqu, which is generally distributed in the central part of the Daqu stack, and its content in HTD is less than 1%, while yellow Daqu, also known as well fermented Daqu, which is evenly distributed throughout the Daqu stack and accounts for 80%–85% of the total HTD (Gan et al., 2019). In actual production, mainly yellow Daqu, supplemented by a small amount of white Daqu and black Daqu, they were mixed and grinded after storage and used for liquor brewing. Hence, it is important to clarify the microbial community structure in the three different types of HTD for liquor production.

In the recent years, with the rapid development of high‐throughput sequencing technology, it has been widely used in analyzing microbial community structure of various environments (Bruno et al., 2017). Compared with traditional microbial culture methods and fingerprint technology, the second‐generation sequencing technology represented by Illumina MiSeq sequencing platform has the advantages of no culture, high‐throughput, fast detection speed, and accurate results (Caporaso et al., 2012; Quail et al., 2012). By adding a nucleotide sequence called a sample‐specific barcode or tag to each sample before sequencing, this technology realizes parallel experiments of multiple samples and solves the disadvantage that traditional fingerprint technology cannot carry out association analysis (King et al., 2014; Roh et al., 2010). In addition, this technology can accurately identify those microorganisms that are difficult to cultivate and have low abundance, as well as achieve a comprehensive, objective, and unbiased revelation of microbial community structure in a microecosystem, providing a new tool for exploring the diversity and function of microorganisms in liquor and Daqu. Up to now, the application of high‐throughput sequencing technology for HTD mainly focused on the effects of different fermentation time (Hu et al., 2017; Su et al., 2015), different regions (Wang, Wu, et al., 2017), different processing methods (Wang et al., 2019), and different environments (Khan et al., 2016) on the microbial community structure of HTD, while the analysis of bacterial diversity in three different types of HTD (white Daqu, black Daqu and yellow Daqu) has rarely been reported.

In this study, 30 HTD of three different types were collected from the same batch with the same HTD‐making process. Through Illumina MiSeq high‐throughput sequencing, the bacterial diversity in different types of HTD was analyzed and compared. Afterward, the bacterial function and phenotype of HTD were predicted based on Phylogenetic Investigation of Communities by Reconstruction of Unobserved States (PICRUSTs) (Douglas et al., 2018) and BugBase (Ward et al., 2017). Therefore, the present study was aimed to (a) fully reveal the bacterial composition of HTD, (b) identify biomarkers to distinguish three different types of HTD, and (c) understand the function as well as phenotype of bacteria in HTD.

## 2 MATERIALS AND METHODS

### 2.1 HTD fermentation and sample collection

The traditional production process of HTD used in this study is shown in Figure 1a. Spontaneous solid‐state fermentation of HTD at an industrial scale was performed in the fermentation chamber of a traditional maotai‐flavor liquor production factory in Xiangyang, Hubei province, China, during June to October (temperature range: 19–34°C), 2019. The HTD‐making process consists mainly of three parts: shaping, fermenting (about 40 d), and ripening (about 180 d). First, raw materials, crushed wheat were mixed with the addition of 8% (w/w) of mother Daqu (HTD produced in the last round), along with 40% (w/w) of water. The mixture was then filled into brick‐shaped molds (37 cm × 18 cm × 7 cm) and the female workers stepped on it with bared feet to obtain sufficient cohesion, allowing the Daqu bricks to retain their shape while maintaining proper air permeability (Figure 1b,c). Second, the resulting Daqu bricks were transferred into fermentation chambers, thereafter layer‐by‐layer stacked up with space between each two, covered with straw, and sprinkled with 1% (w/w) of water, to create a proper fermentation environment for microorganisms; when the Daqu‐brick temperature reached around 65°C, Daqu bricks are rearranged to adjust the temperature and humidity, letting each brick fermenting evenly. After 40 d of fermentation, when the Daqu‐brick temperature was close to room temperature, the workers dismantle Daqu‐brick walls and all Daqu bricks are stored for ripening in the open air for about 180 days, and HTD were subsequently obtained.

In this study, 30 samples were collected from the HTD finished products, including 10 each for white Daqu, black Daqu, and yellow Daqu, using a fully randomized experimental design. All HTD samples were made in the same batch and under the same conditions adopting the traditional HTD‐making process. The samples were ground to powder in an alcohol‐disinfected grinder, collected into standard sampling tubes with sterile scoops, then placed in a low‐temperature sampling box, and transported back to the laboratory within 24 hr for storage at −20°C.

### 2.2 Metagenomic DNA extraction

The metagenomic DNA was extracted from 2.0 g of each sample using the QIAGEN DNeasy mericon Food Kit according to the manufacturer's instructions (QIAamp DNA Microbiome Kit, QIAGEN Inc.). The purity, concentration, and integrity of the extracted DNA were detected by spectrophotometry and 1% agarose gel electrophoresis. Qualified DNA samples were stored in a −20°C refrigerator for use.

### 2.3 PCR amplification and MiSeq high‐throughput sequencing

In this study, the V_3_‐V_4_ region of 16S rRNA was amplified using forward primer 338F (5'‐ACTCCTACGGGAGGCAGCAG‐3') and reverse primer 806R (5'‐GGACTACHVGGGTWTCTAAT‐3') (Xu et al., 2016). The PCR amplification system included the following: 4 μl 5 × PCR buffer, 2 μl 2.5 mM dNTP mix, 0.8 μl 5 μmol/l forward primer, 0.8 μl 5 μmol/l reverse primer, 0.4 μl 5 U/μl DNA polymerase, 10 ng DNA template, and supplemented to 20 μl with ddH_2_O. The PCR amplification conditions were as follows: 95°C for 3 min; 95°C for 30 s, 55°C for 30 s, 72°C for 45 s, 30 cycles; and 72°C for 10 min.

The 30 qualified DNA amplicons, diluted to a concentration of 100 nmol/l, were sequenced by an MiSeq high‐throughput sequencing platform in Shanghai Majorbio Co., Ltd.

### 2.4 Quality control of the sequences

The pair‐ended sequences generated through MiSeq sequencing were merged referring to the overlap relationship of paired fragment sequences. Data were selected using the following criteria: the base demand of overlap area greater than 10 bp; the maximum mismatch ratio ≤ 0.2; the assembled sequence has no barcode base mismatch; the primer base mismatch number ≤ 2 bp; sequences with read length longer than 475 bp. The qualified assembly was classified to different samples by referring to the barcode. The barcode and primers in the aligned sequences were removed to obtain high‐quality information for further analysis. Sequences with less than 50 bp of bases after cutting were discarded.

### 2.5 Bioinformatics analysis

Species analysis and diversity assessment were performed on the retained sequences using the QIIME (v1.90) platform. The procedure was as follows: (a) calibrating and aligning the high‐quality sequences with PyNAST (Caporaso et al., 2010); (b) using UCLASS (Edgar, 2010) to cluster the aligned sequences based on 100% similarity to establish a complete representative gene set of 16S rRNA; (c) clustering the aligned sequences based on 97% similarity by UCLASS to generate operational taxonomic units (OTUs) (Hou et al., 2016); (d) identifying and deleting chimeras in the constructed OTU matrix through ChimeraSlayer (Wei et al., 2016); (e) performing homology comparison among the representative reads with RDP Release, Greengenes, and SILVA databases, annotating taxonomic positions at the phylum, class, order, family, genus, and species levels, and combining annotation results generated by different databases (Balvočiūtė & Huson, 2017); (f) drawing phylogenetic tree based on OTU representative sequence with FastTree (Yu et al., 2017); (g) assessing bacterial abundance and diversity in each sample basing on α diversity indexes including the observed species, Shannon index, Chao1 index, and Simpson index, evaluating whether the sequencing depth meets the requirements of subsequent bioinformatics analysis with Shannon index curve and exponential curve of observed species; (h) leveling all samples 1,000 times randomly based on the OTU matrix data according to the number of samples containing the minimum sequences to reduce error, combining the results obtained from the 1,000 replicates, and doing β diversity analysis; (i) investigating intersample β diversity by principal coordinates analysis based on Bray–Curtis distance and permutational multivariate analysis of variance.

### 2.6 Prediction of microbiome phenotypes and bacterial potential functional

The remaining sequences were clustered into OTU matrix by UCLASS with the identity threshold of 97% according to the standard database of PICRUSTs. Taxonomic positions of representative sequences were annotated with the Greengenes database. The PICRUSt software was employed to predict the functional potential of the bacterial communities in HTD samples. At the same time, the annotated OTU matrix was uploaded to the online Web site (https://bugbase.cs.umn.edu/) for microbiome phenotype prediction (Ward et al., 2017).

### 2.7 Statistical analysis

Principal coordinates analysis (PCoA) was conducted with R software (version 3.6.3, https://www.r‐project.org/). While permutational multivariate analysis of variance (PERMANOVA) and multivariate analysis of variance (MANOVA; Weinfurt, 2010) were calculated by the data processing system (DPS; version 9.50, http://www.chinadps.net/; Tang & Zhang, 2013). Linear discriminant analysis effect size (LEfSe) algorithm was applied with the online interface utilizing the Huttenhower Lab Galaxy Server (http://huttenhower.sph.harvard.edu/galaxy/). Figures were plotted mainly using R software and Origin (version 2018, Origin Lab).

## 3 RESULTS

### 3.1 Analysis of bacterial diversity in different types of HTD samples

Using the Illumina MiSeq system, 1,385,378 filtered 16S rRNA gene sequences were obtained from 30 HTD samples, and an average of 46,179 sequences were generated per sample (range: 17,942–65,067, *SD* = 9,775). According to the 97% similarity threshold, sequences were divided and 5,234 OTUs were obtained after removal of chimeric OTUs, and the average number of OTUs obtained by each sample was 702 (range: 674–1,001, *SD* = 185). Statistics analysis of the sequencing information depicts the average number of reads, and OTUs were 48,816 (range: 41,448–65,067, *SD* = 7,228) and 711 (range: 360–1,001, *SD* = 191), respectively, for the 10 white Daqu; 46,267 (range: 35,061–62,233, *SD* = 7,360) and 710 (range: 270–906, *SD* = 185), respectively, for the 10 black Daqu, while 43,455 (range: 17,942–62,965, *SD* = 13,619) and 686 (range: 303–960, *SD* = 198), respectively, for the 10 yellow Daqu. The 16S rRNA sequencing results of HTD samples and the number of each taxonomic position are shown in Table S1

The sparse curve and the Shannon index curve were used to further evaluate the existing sequencing depth to determine whether it meets the requirements of subsequent bioinformatics analysis. From Figure S1, the OTU number increases with the depth of sequencing, and Shannon index curve also increases continuously. With the gradual increase in sequencing depth, the number of OTUs did not reach equilibrium, but there was still an upward trend, while Shannon index curve of all samples has entered or approached the plateau stage. This signifies that although new OTUs and bacterial species may be discovered by expanding the coverage, the current sequencing depth has covered most of the bacterial diversity in the sample, which is sufficient to fully display the bacterial abundance information in the sample and meet the requirements of subsequent bioinformatics analysis.

**FIGURE 2:**
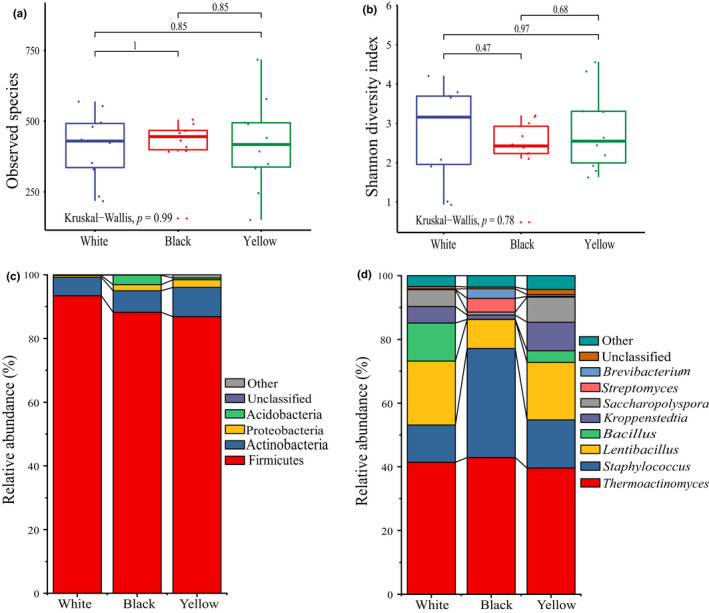
Boxplots of α‐diversity indexes (a and b) and relative abundance and bacterial diversity of HTD samples at the level of phylum and genus (c and d). (a) Number of observed species; (b) Shannon index. The whisker caps represent the minimum and maximum values. The box plot middle, upper, and lower lines represent the median value and first and third quartiles, respectively

To compare the bacterial richness and diversity of three different types of HTD, the bacterial data set of HTD samples was analyzed. As shown in Figure 2a,b, the number of observed species and Shannon index of the three different types of HTD were not significantly different (*p* > .05), illustrating that there was no significant difference (*p* > .05) among them in bacterial richness and diversity.

### 3.2 Analysis of bacterial community structure in different types of HTD samples

In this study, bacterial phyla or genera with an average relative abundance of more than 1.00% were defined as dominant phyla or genera, other phyla or genera with less than 1.00% were classified as others, and sequences that could not be identified at the phylum or genus level were classified as unclassified.

A total of seven bacterial phyla were identified from all HTD samples, and four were the dominant bacterial phyla among them, namely Firmicutes (89.49%), Actinobacteria (7.26%), Proteobacteria (1.62%), and Acidobacteria (1.21%), which accounted for 99.59% of the total sequences (Figure 2c,d). The average relative abundance of Firmicutes in all 30 HTD samples was > 43.79%, and this made it an absolute dominant bacterial phylum. Actinobacteria were also present in all HTD samples, although its abundance in some samples was low. At the genera level, a total of 262 bacterial genera were identified (Figure 2d), and eight were the dominant bacterial genera among them, namely *Thermoactinomyces* (41.30%), *Staphylococcus* (20.36%), *Lentibacillus* (15.73%), *Bacillus* (5.21%), *Kroppenstedtia* (5.14%), *Saccharopolyspora* (4.72%), *Streptomyces* (1.53%), and *Brevibacterium* (1.23%), which accounted for 95.23% of the total sequences. Besides, *Thermoactinomyces*, *Staphylococcus*, *Lentibacillus*, *Kroppenstedtia,* and *Saccharopolyspora* were present in all HTD samples, making them the absolute dominant bacterial genera.

**FIGURE 3:**
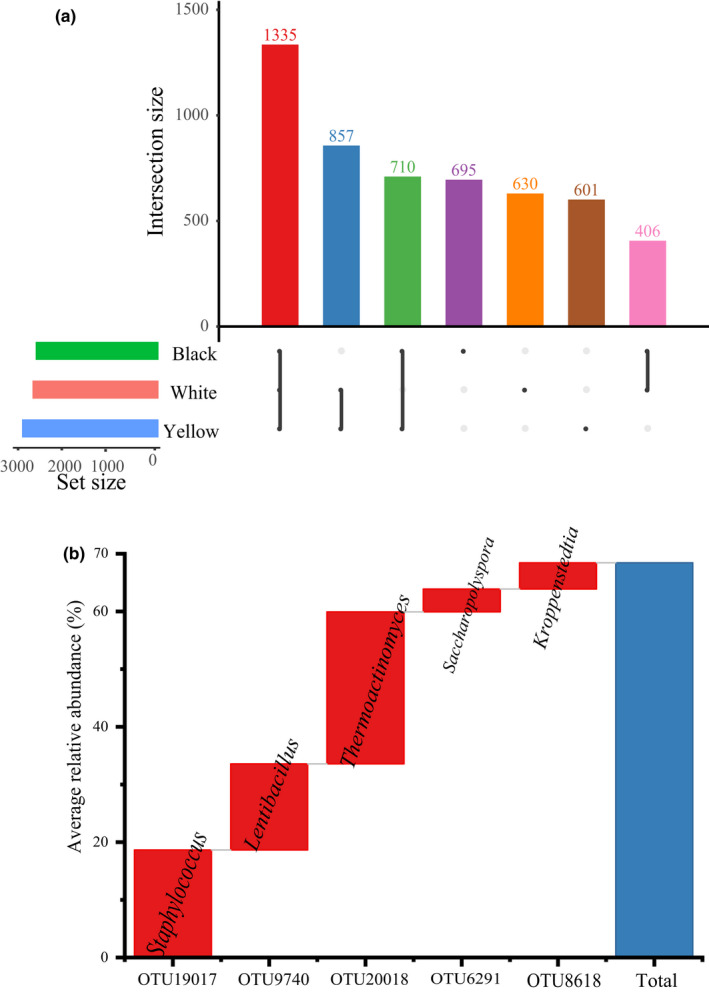
Relative abundance and bacterial diversity of HTD samples at OTU level

The bacterial community structure in three different types of HTD was further analyzed at the OTU level (Figure 3). Of the 5,234 OTUs found in all HTD samples, 1,335 were present in all three types of HTD (Figure 3a). Among them, there are five core OTUs, which are present in all Daqu samples with relative abundance > 1.00%, including OTU19017 (*Staphylococcus*, 18.64%), OTU9740 (*Lentibacillus*, 14.93%), OTU20018 (*Thermoactinomyces*, 26.36%), OTU6291 (*Saccharopolyspora*, 3.97%), and OTU8618 (*Kroppenstedtia*, 4.56%; Figure 3b). The cumulative relative abundance of these five core OUTs was as high as 68.45% and contained 936,719 sequences, which accounted for 68.60% of the total sequences, suggesting that there were a large number of core bacterial communities in three different types of HTD, and they were mainly subordinate to the five genera including *Staphylococcus*, *Lentibacillus*, *Thermoactinomyces*, *Saccharopolyspora,* and *Kroppenstedtia*.

### 3.3 Different analysis of bacterial community structure in different types of HTD samples

**FIGURE 4:**
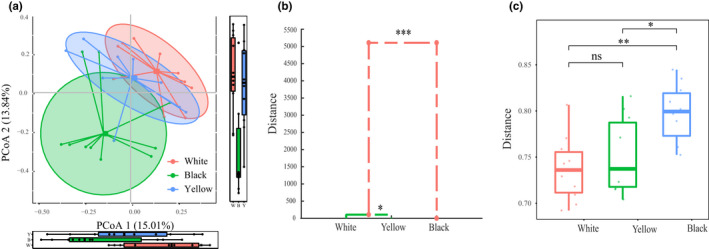
PCoA score plots based on Bray–Curtis distance (a); dendrogram based on Bray–Curtis distance calculated using Mahalanobis distances as well as MANOVA (b); within‐group variations of the 3 different types of HTD using Bray–Curtis distance (c). Significant difference is represented by *** (*p* < .001), ** (*p* < .01), * (*p* < .05) and ns (*p* ≥ .05), respectively

Principal coordinates analysis, MANOVA, and analysis of within‐group variations were applied to visualize the bacterial community structure differences in three different types of HTD samples (Figure 4). PCoA based on Bray–Curtis distance shows that although 3 different types of HTD samples have some overlaps in spatial arrangement, their clustering trend is still obvious (Figure 4a). PERMANOVA further proves that there were extremely significant differences in bacterial community structure among three different types of HTD samples (*p* = .001). It is worth noting that, in the PCoA score plot, the spatial distance between white Daqu samples and yellow Daqu samples is closer than that of black Daqu samples, implying that the bacterial community structure of white Daqu samples and yellow Daqu samples may be more similar, while that of black Daqu samples differs more from the two.

Meanwhile, data obtained from the PCoA were evaluated by MANOVA, a constrained classification of feature vectors into clusters via Mahalanobis distances, to categorize the three different types of HTD samples based on Bray–Curtis distance. The dendrogram generated from MANOVA providing a clear visualization of the relationships among the three different types of HTD is shown in Figure 4b. Significant difference (*p* < .05) was observed in bacterial community structure of 3 different types of HTD samples. Below the average distance of 500, white Daqu and yellow Daqu cluster; then above the average distance of 5,000, black Daqu cluster with white Daqu and yellow Daqu to become a group. Moreover, the difference between black Daqu and the cluster of white Daqu along with yellow Daqu was extremely significant (*p* = 8.92*10^–10^ < 0.01), while the difference between white Daqu and yellow Daqu was significant (*p* = .03 < 0.05), proving the bacterial community structure of white Daqu samples was more similar to that of yellow Daqu samples rather than that of black Daqu samples. This also confirms the results in PCoA.

Furthermore, within‐group variations among the three different types of HTD samples were calculated using Bray–Curtis distance. It is shown in Figure 4c that the within‐group variation of white Daqu samples and black Daqu samples is significantly lower (*p* < .05) than that of yellow Daqu samples; meanwhile, no significant differences (*p* > .05) of the within‐group variation between white Daqu samples and black Daqu samples were found. Remarkably, yellow Daqu samples have the highest within‐group variation, demonstrating that the bacterial community structure of white Daqu samples and black Daqu samples was more stable, while that of yellow Daqu samples was most different. This implies that yellow Daqu might provide more possibilities for bacterial diversity in maotai‐flavor liquor fermentation, which could be meaningful both theoretically and practically.

**FIGURE 5:**
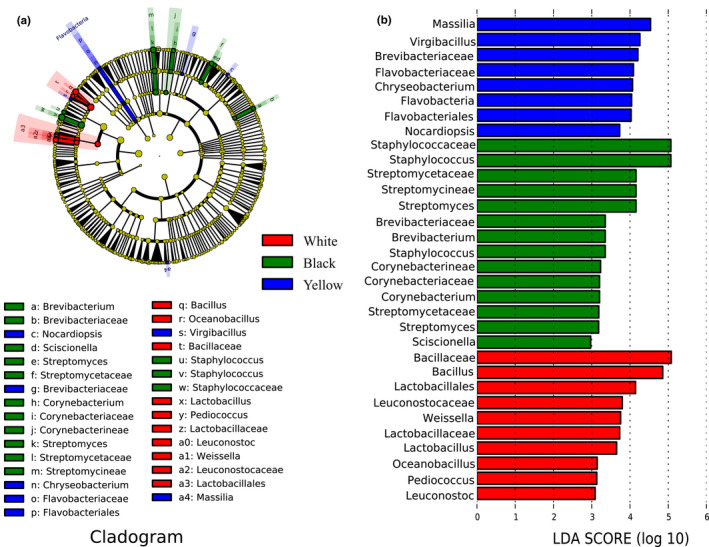
Identification of discriminant taxa between 3 different types of HTD by LDA of the effect size. Cladogram of the microbiota (a). Significant discriminant taxon nodes of white Daqu, black Daqu, and yellow Daqu are represented by red, green, and blue, respectively, while nondiscriminant taxon nodes are represented by yellow. Branch areas are shaded according to the highest ranked variety for that taxon. The LDA score indicates the level of differentiation among different types of HTD. A threshold value of 2.0 was used as the cutoff level. Horizontal bar chart showing discriminant taxa (b). Significant discriminant taxa of white Daqu, black Daqu, and yellow Daqu are represented by red, green, and blue, respectively

To sum up, there were significant differences in the bacterial community structure in different types of HTD samples (*p* < .05). In order to identify the difference in bacterial community structure, LEfSe was performed with an LDA threshold score of 3.0 (Figure 5). A total of 32 differential taxa with significantly different abundance (*p* < .05) were identified among all HTD samples, of which 10, 14, and 8 had significant effects (*p* < .05) on white Daqu, black Daqu, and yellow Daqu, respectively. Then, LEfSe further pinpointed the differential taxa in these bacterial communities. The results indicate that *Bacillus* could be used as a biomarker in white Daqu, and *Staphylococcus* could be used as a biomarker in black Daqu, while *Massilia* could be used as a biomarker in yellow Daqu (Segata et al., 2016).

### 3.4 Prediction of bacterial functional potential and microbiome phenotypes

**FIGURE 6:**
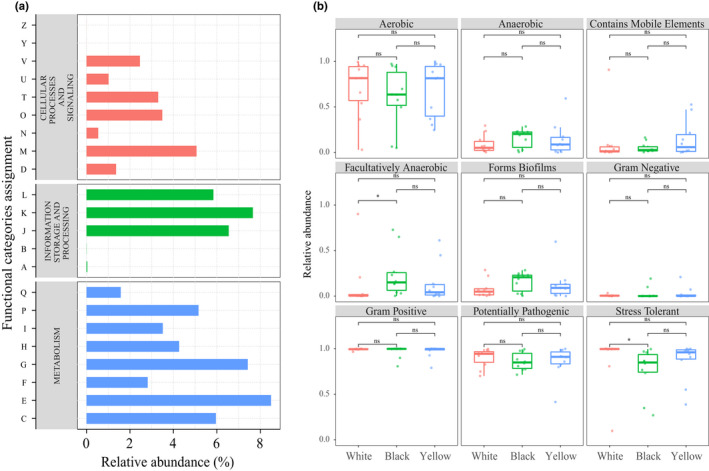
Overview of the bacterial COG profile (a), COG functional categories codes are as follows: A = RNA processing and modification; B = chromatin structure and dynamics; C = energy production and conversion; D = cell cycle control, cell division, chromosome partitioning; E = amino acid transport and metabolism; *F* = nucleotide transport and metabolism; G = carbohydrate transport and metabolism; H = coenzyme transport and metabolism; I = lipid transport and metabolism; J = translation, ribosomal structure, and biogenesis; K = transcription; L = replication, recombination, and repair; *M* = cell wall/membrane/envelope biogenesis; *N* = cell motility; O = posttranslational modification, protein turnover, chaperones; P = inorganic ion transport and metabolism; Q = secondary metabolite biosynthesis, transport, and catabolism; R = general function prediction only; S = function unknown; T = signal transduction mechanisms; U = intracellular trafficking, secretion, and vesicular transport; V = defense mechanisms; W = extracellular structures; Y = nuclear structure; Z = cytoskeleton. Comparative analysis of microbiome phenotypic results of HTD samples (b). Significant difference is represented by * (*p* < .05) and ns (*p* ≥ .05), respectively

To better understand the role of bacteria in HTD samples, based on high‐throughput sequencing data of 16S rRNA, PICRUSt was employed to predict the functional potential of bacteria in HTD samples further analysis was carried out in the context of the Cluster of Orthologous Groups (COG) database. Results a total of 4,794 functional proteins or enzymatic COGs belonging to 22 COG functional categories were identified and a bacterial COG profile was obtained. Enrichment of functions of metabolism in HTD samples can be observed in Figure 6a, which means that the bacterial metabolism in HTD is intense. Through Kruskal–Wallis rank‐sum test (Figure S2), among the 22 COG functional categories, only a significantly difference (*p* < .05) in category Z (cytoskeleton) with the lowest functional abundance was obtained. It can also be observed from Figure 6a that categories E (amino acid transport and metabolism), K (transcription) along with G (carbohydrate transport and metabolism) dominated in all HTD samples, and the relative abundance of functional category E was the highest among all functional categories.

The potential prediction for phenotypic functions of bacteria in three different types of HTD samples detected nine potential microbial phenotypes including aerobic, anaerobic, contains mobile elements, biofilm forming, facultatively anaerobic, forms biofilms, Gram‐negative, Gram‐positive, potentially pathogenic, and stress tolerant (Figure 6b). The differences in relative abundance of two predicted phenotypic functions (Facultatively Anaerobic and stress tolerant) were significant (*p* < .05), while the rest were not significant (*p* > .05).

## 4 DISCUSSION

High‐temperature Daq is produced by traditional spontaneous solid‐state fermentation in an open‐work environment (Huang et al., 2017). The fermentation process for HTD involves abundant microorganisms under high‐temperature conditions, which is quite different from other types of Daqu. Bacteria are important microorganisms producing various enzymes and aroma substances in HTD. In the present study, Illumina MiSeq high‐throughput sequencing technology was applied to analyze the bacterial diversity of white, black, and yellow HTDs, respectively. It was found that the absolute dominant bacterial genera in HTD were *Thermoactinomyces*, *Staphylococcus*, *Lentibacillus*, *Kroppenstedtia,* and *Saccharopolyspora* (Figure 2). Different from the results of this study, Jin et al. (2019) used high‐throughput sequencing technology and revealed dominant bacteria in HTD of Moatai town and Xijiu town, Guizhou province, China, were Bacillales, Enterobacteriales, and Lactobacillales. This demonstrates that HTD in different regions has a unique bacterial community structure due to environmental differences. Among the five absolute dominant bacterial genera in the present study, *Thermoactinomyces* has the highest relative abundance as well as strong heat resistance ability, which is often detected in HTD or medium‐high‐temperature Daqu, and has the potential function of providing rich flavor substances and flavor precursors (Yoon & Park, 2000). For example, *Thermoactinomyces vulgaris* and *Thermoactinomyces sacchari*, which are subordinate to *Thermoactinomyces*, not only have the ability to secrete amylase, esterase, cellulase, pectinase, and phosphatase, but also can produce pyrazine substances with maotai‐flavor and other flavor substances, which are considered to be crucial in Chinese liquor brewing (Xiao et al., 2017; Zheng et al., 2015).

Principal coordinates analysis and MANOVA (Figure 3) showed that there were significant differences (*p* < .05) in the bacterial community structure of the three different types of HTD, among which the bacterial community structure of black Daqu was significantly different from that of the other two HTDs. This implies that the bacterial community structure of black Daqu may be more unique than that of white Daqu and yellow Daqu. Interestingly, it is found that the average relative abundance of *Streptomyces* was only 0.07% and 0.37% in white Daqu and yellow Daqu, respectively, while it reached 4.27% in black Daqu, and the difference between them was extremely significant (*p* < .01). Previous study found that *Streptomyces* originated from the air in the production room during liquor production and have the ability to lipid esterase, secrete esterase, alkaline phosphatase along with phosphate hydrolase, which may play an important role in the formation of flavor substances or flavor precursors in maotai‐flavor liquor (Zheng et al., 2015).

The results of LEfSe (Figure 5) demonstrated that the biomarker in white Daqu was *Bacillus*, and its relative abundance was significantly higher (*p* < .05) than that in black Daqu as well as yellow Daqu. *Bacillus* has been previously reported as the one of the most represented and most important bacteria in multiple types of Daqu (Hu et al., 2017; Zhang et al., 2014; Zheng et al., 2014). *Bacillus* has also been proved to be the main maotai‐flavor‐producing functional bacteria in the production of maotai‐flavor liquor and has strong ability to secrete a wide range of degrading enzymes including protease, amylase, cellulase and glucoamylase, which can hydrolyze macromolecular substances such as protein and starch to metabolize and produce numerous flavor compounds during the fermentation process of maotai‐flavor liquor (Hoshino & Morimoto, 2008; Kong et al., 2014; Li, Lian, et al., 2014; Li, Bai, et al., 2014; Su et al., 2015; Yan et al., 2015; Zhang et al., 2013; Zhao et al., 2017; Zheng et al., 2012, 2013). The type and quantity of *Bacillus* bacteria directly affect the quality of Daqu and determine the difference between liquor quality along with style (Fan et al., 2018). For instance, *Bacillus licheniformis* was found to produce more than 70 metabolites, and it could exhibit strong enzymatic activity with respect to the proteases, lipase, thermostable α‐amylase, cellulase, and hemicellulose as well as produce aromatic compounds, C4 compounds, pyrazines, and organic acids by solid‐state fermentation (Li, Bai, et al., 2014; Wu et al., 2014; Yan et al., 2013; Zhang et al., 2013; Zheng et al., 2013). *Bacillus cereus* could produce ethyl hexanoate, an important flavor compound in Chinese Baijiu (Zhao et al., 2017). While *Bacillus amyloliquefaciens* and *Bacillus subtilis* were reported to be the flavor producing strains, they can secrete α‐amylase to make sugar in raw materials ferment quickly and become fully decomposed into alcohols, esters along with various organic acids (Li, Bai, et al., 2014; Su et al., 2015). He et al. (2019) found that the relative abundance of *Bacillus* in Daqu was higher at sites with higher oxygen content. For this reason, the biomarker in white Daqu, which is distributed in the top layer of Daqu stacks and is more sufficiently exposed to oxygen, is *Bacillus*. As for the black Daqu which is distributed in the central part of the Daqu stacks with the worst air permeability and the least oxygen contact, its relative abundance of *Bacillus* is certainly the lowest. What's more, *Bacillus* is capable of causing strong stress tolerance to various environment factors including heat, acid, and oxygen owing to the spore formation (Lay et al., 2015; Wang et al., 2018; Xiong et al., 2014). This also explains why white Daqu with the highest relative abundance of *Bacillus* had significantly higher (*p* < .05) functional abundance on phenotypic functions of stress tolerant than black Daqu with the lowest relative abundance of *Bacillus* (Figure 5b). In addition, *Bacillus* was found to be the dominant bacterial genus in wheat (Zheng et al., 2015). Thus, this illustrates that raw materials act as important sources of microorganisms in HTD (Du et al., 2019).

The biomarker in black Daqu was *Staphylococcus*, which was also one of the absolute dominant bacterial genera in HTD. It is widely distributed in the natural environment, and some members of *Staphylococcus* have been involved in human infections as opportunistic pathogens (Zhang et al., 2018). For example, *Staphylococcus aureus*, which subordinate to *Staphylococcus*, is the most common pathogenic bacteria in human suppurative infections and can cause contamination of food during processing, storage, and transportation (Tong et al., 2015). Stevens et al. (2015) utilized microbiomic analysis and discovered that *Staphylococcus* were dominant across different foot sites and comprised almost the entire bacterial population on the plantar surface. Du et al. (2019) applied high‐throughput sequencing technology combined with microbial source tracking analysis and proved that both the raw materials and the processing environments act as important sources for Daqu microorganisms. It follows that due to the relatively open processing environment, especially the barefoot stepping process by female workers, there may be a small number of harmful microorganisms or spoilage bacteria originating from the environment or female workers' feet in HTD. Therefore, it is particularly important to optimize the HTD processing environment.

Notably, *Massilia* was found to be the biomarker in yellow Daqu. *Massilia* is widely distributed in soil (Feng et al., 2016), air (Orthova et al., 2015), water (Gallego et al., 2006), and other environments. *Massilia* has exhibited a high degree of adaptability to temperature, and different *Massilia* strains can grow in the temperature range of 0–55°C (Xu et al., 2005). It is speculated that *Massilia* in yellow Daqu originated mainly from the processing environment and survived during the HTD‐making process. *Massilia* can not only synthesize a variety of secondary metabolites and enzymes, but also utilize starch, maltotriose, or maltose as sole carbon source to ferment (Han et al., 2014). However, compared with species and strains of other genera, the species and strains found in *Massilia* are still very limited, with great potential for exploitation. The function and application of *Massilia* in fermented food such as liquor need to be further clarified.

It is worth mentioning that six genera of lactic acid bacteria were detected in HTD samples, including *Lactobacillus* (0.36%), *Weissella* (0.35%), *Pediococcus* (0.11%), *Enterococcus* (0.02%), *Leuconostoc* (0.05%), and *Streptococcus* (0.00%). Although these lactic acid bacteria were found in low abundance, LEfSe (Figure 4) still showed that the relative abundance of lactic acid bacteria in white Daqu was significantly higher (*p* < .05) than that in both black Daqu and yellow qu. Lactic acid bacteria were reported to be important during the production of other types of Daqu, since it can not only synthesize exopolysaccharides and oligosaccharides together with the Maillard reaction in the formation of aroma substances of liquor, but also produce generous lactic acid, which is the precursor of ethyl lactate, and can enhance the mellowness along with sweetness of liquor (Gao et al., 2014; He et al., 2019; Su et al., 2015; Xie et al., 2020; Yang et al., 2019; Zheng et al., 2012, 2015). Zheng et al. (2015) observed that a high abundance of lactic acid bacteria only exists in the early stages of HTD production. However, the increase in temperature in the whole fermentation process leads to the rapid growth of thermophilic bacteria such as *Thermoactinomyces* and *Bacillus* in HTD samples and the slower growth of mesophilic lactic acid bacteria (Li et al., 2017). Moreover, *Bacillus*, the dominant bacterial genus in HTD, has also been proved to have the ability to inhibit the growth of lactic acid bacteria (Zhao et al., 2020). This might explain the low abundance of lactic acid bacteria in the HTD samples. Besides, compared with the other two types of HTD, white Daqu was distributed in the top layer of the Daqu stack and its heat dissipation and air permeability were better, which leads to a relatively higher abundance of lactic acid bacteria.

The results of bacterial functional potential prediction and microbiome phenotype prediction show that most of the bacterial functional potential and microbiome phenotypes were not significantly different (*p* > .05) among different types of HTD, indicating bacteria in different types of HTD have similar functions and phenotypes. The predicted bacterial COG profile functional profiles by PICRUSt exhibited the highest relative abundance of functional category E (amino acid transport and metabolism) among all functional categories, suggesting that the transport and metabolism of amino acids in HTD dominate. At the same time, the transport and metabolism of amino acids also requires energy. In HTD, amino acids participated in Maillard reaction at high temperature, which led to the change in HTD color and endowed HTD with unique maotai‐flavor (Gan et al., 2019). The prominent expression of functional category G (carbohydrate transport and metabolism) provides a large amount of energy for the growth and reproduction of microorganisms, material transport, and respiration. Amino acid metabolism and carbohydrate metabolism are essential for microorganisms and play an important role in the survival of microorganisms and the exercise of their related functions. Simultaneously, it is also found that the HTD samples also accounted for a considerable relative abundance in the functional category J (translation, ribosomal structure, and biogenesis), L (replication, recombination, and repair), and *M* (cell wall/membrane/envelope biogenesis), showing a high degree of translation, modification, and conversion, which was indicative of the frequent metabolism in the HTD samples, representing that the bacteria in the HTD maintained a high growth and reproductive capacity. Furthermore, the HTD samples also had a high relative abundance on functional category P (inorganic ion transport and metabolism) and H (coenzyme transport and metabolism). Transport of various ions is also necessary for bacterial metabolism, which can act as cofactors for various enzymes, thereby promoting a wide variety of biochemical reactions. As for coenzyme transport and metabolism, the enzymes in HTD have been proved not only to catalyze the metabolism of amino acids to accelerate the fermentation rate in HTD, but also to provide substrates and conditions for Maillard reaction under high‐temperature environment, causing browning and maotai‐flavor of HTD (Gan et al., 2019; Xie et al., 2020).

## 5 CONCLUSIONS

There are abundant and complex bacterial communities with vigorous bacterial metabolism in HTD, and the bacterial community structure of different types of HTD is significantly diverse (*p* < .05). Due to the production of HTD is processed in an open environment with high‐temperature, the composition of bacterial community structure in HTD is affected by this special environment. The dominant bacteria in HTD are mainly derived from 3 aspects: thermophilic bacteria (such as *Thermoactinomyces*), dominant bacteria in raw materials (such as *Bacillus*), and dominant bacteria in processing environment (such as *Staphylococcus*). Further studies are required to identify the specific environmental factors in HTD that contribute to the diversity in bacterial community structure and quality of different types of HTD. This work may provide a scientific basis for the rational implementation of microbial control and enhancement of the production process for maotai‐flavor liquor in the future.

## CONFLICT OF INTEREST

The authors declare no conflict of interest.

## Supporting information

Figure S1

Figure S2

Table S1

## Data Availability

The data that support the findings of this study are publicly available for scientific research, and the links available for the sequences involved in this paper are as follows: https://www.mg‐rast.org/mgmain.html?mgpage=metazen2&project=mgp96594.
