# Supplementary figures and images for: Analysis of microbial diversity and functional differences in different types of high‐temperature Daqu
Source: Food Sci Nutr. 2020 Dec 17;9(2):1003–16. doi: 10.1002/fsn3.2068 (PMC7866569; doi:10.1002/fsn3.2068)

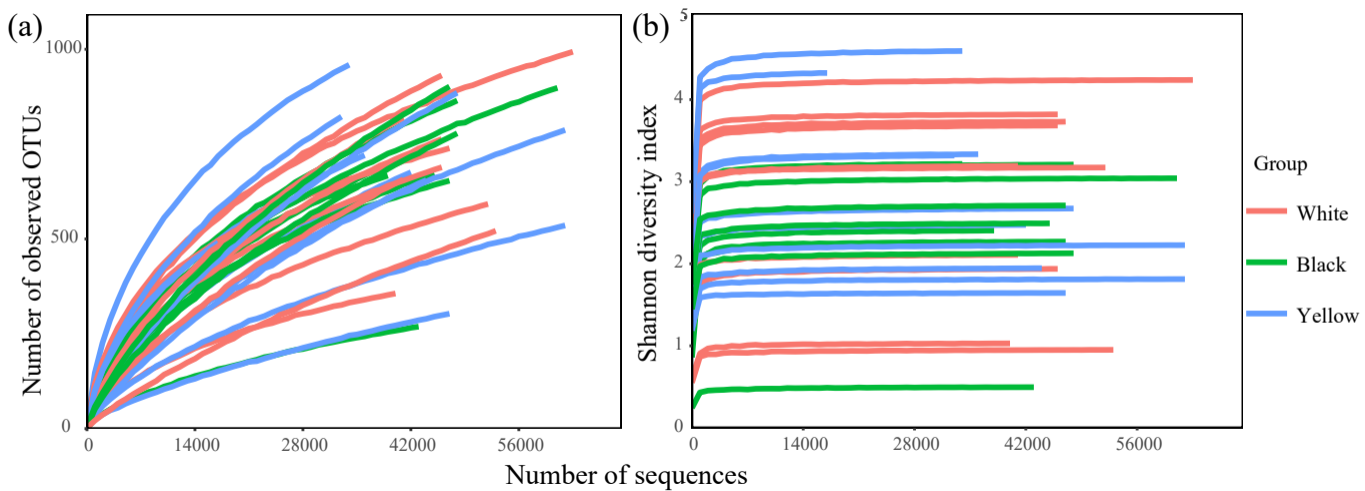

Supplement: Supplementary file 1 — Figure S1 [file FSN3-9-1003-s001.pdf]

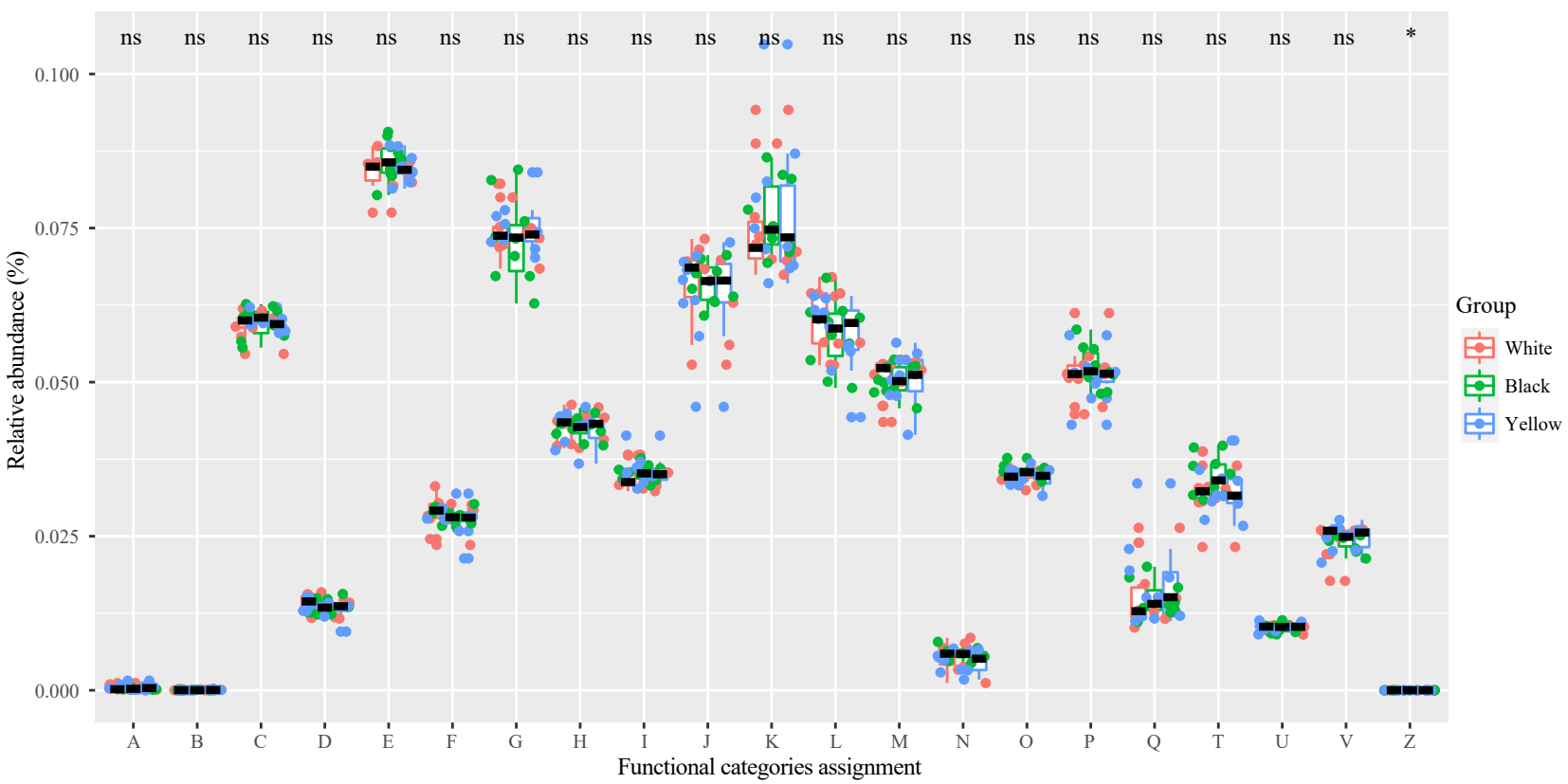

Supplement: Supplementary file 2 — Figure S2 [file FSN3-9-1003-s002.pdf]
