# Supplementary material for: Analysis of microbial diversity and functional differences in different types of high‐temperature Daqu
Source: Food Sci Nutr. 2020 Dec 17;9(2):1003–16. doi: 10.1002/fsn3.2068 (PMC7866569; doi:10.1002/fsn3.2068)
Supplement: Supplementary file 3 — Table S1 [file FSN3-9-1003-s003.docx]

**TABLE S1** General information of sequence and alpha diversity in HTD samples

| Samples | Number of reads | Number of OTU | Chao1 index | observed species | shannon index | simpson index |
| --- | --- | --- | --- | --- | --- | --- |
| White1 | 41448 | 360 | 429 | 234 | 1.02 | 0.19 |
| White2 | 47503 | 695 | 787 | 424 | 3.80 | 0.87 |
| White3 | 54101 | 524 | 886 | 218 | 0.94 | 0.24 |
| White4 | 42200 | 827 | 1155 | 481 | 3.16 | 0.75 |
| White5 | 47480 | 940 | 1107 | 554 | 3.66 | 0.79 |
| White6 | 48473 | 744 | 790 | 496 | 3.71 | 0.81 |
| White7 | 46976 | 767 | 898 | 435 | 1.91 | 0.45 |
| White8 | 65067 | 1001 | 969 | 570 | 4.21 | 0.87 |
| White9 | 41563 | 658 | 1090 | 353 | 2.08 | 0.56 |
| White10 | 53348 | 595 | 657 | 330 | 3.15 | 0.78 |
| Average+SD | 48816±7228 | 711±191 | 877±224 | 410±123 | 2.76±1.19 | 0.63±0.26 |
| Black1 | 62233 | 901 | 935 | 468 | 3.01 | 0.72 |
| Black2 | 39536 | 697 | 959 | 432 | 2.39 | 0.62 |
| Black3 | 48729 | 780 | 1073 | 396 | 2.10 | 0.60 |
| Black4 | 48420 | 659 | 737 | 409 | 2.25 | 0.44 |
| Black5 | 49820 | 873 | 1010 | 506 | 3.18 | 0.76 |
| Black6 | 47954 | 906 | 1288 | 459 | 2.68 | 0.72 |
| Black7 | 40716 | 674 | 766 | 467 | 2.23 | 0.40 |
| Black8 | 35061 | 664 | 822 | 491 | 3.20 | 0.73 |
| Black9 | 46108 | 678 | 823 | 393 | 2.47 | 0.65 |
| Black10 | 44089 | 270 | 365 | 157 | 0.49 | 0.09 |
| Average+SD | 46267±7360 | 710±185 | 878±244 | 418±100 | 2.4±0.78 | 0.57±0.21 |
| Yellow1 | 47727 | 303 | 488 | 151 | 1.63 | 0.57 |
| Yellow2 | 62965 | 788 | 880 | 350 | 2.20 | 0.65 |
| Yellow3 | 62727 | 536 | 593 | 246 | 1.80 | 0.50 |
| Yellow4 | 45372 | 662 | 1064 | 334 | 1.93 | 0.58 |
| Yellow5 | 37081 | 724 | 905 | 496 | 3.31 | 0.75 |
| Yellow6 | 34896 | 960 | 1162 | 718 | 4.56 | 0.84 |
| Yellow7 | 42870 | 678 | 994 | 394 | 2.45 | 0.63 |
| Yellow8 | 49307 | 890 | 1268 | 441 | 2.64 | 0.67 |
| Yellow9 | 17942 | 494 | 764 | 491 | 4.32 | 0.88 |
| Yellow10 | 33666 | 824 | 1144 | 580 | 3.30 | 0.74 |
| Average+SD | 43455±13619 | 686±198 | 926±253 | 420±164 | 2.82±1.03 | 0.68±0.12 |

*Note:* The alpha diversity index values in the table are all obtained when the sequencing depth is 17010.
